# Heart Failure Care Facilitators and Barriers in Rural Haiti: A Qualitative Study

**DOI:** 10.5334/aogh.4521

**Published:** 2024-09-25

**Authors:** Gene F. Kwan, Elizabeth Basow, Benito D. Isaac, Darius L. Fenelon, Evyrna Toussaint, Dawson Calixte, Michel Ibrahim, Lisa R. Hirschhorn, Mari-Lynn Drainoni, Alma Adler, Mary A. Clisbee, Gene Bukhman

**Affiliations:** 1 Section of Cardiovascular Medicine, Department of Medicine, Boston Medical Center & Boston University Chobanian & Avedisian School of Medicine, Boston, MA, USA; 2 Partners In Health, Boston, MA, USA; 3 Boston University Chobanian & Avedisian School of Medicine, Boston, MA, USA; 4 Zanmi Lasante, Haiti; 5 Boston University School of Public Health, Boston, MA, USA; 6 Section of Cardiovascular Medicine, Boston University Chobanian & Avedisian School of Medicine, Boston, MA, USA; 7 Ryan Family Center on Global Primary Care, Robert J Havey Institute for Global Health, Northwestern University Feinberg School of Medicine, Chicago, IL, USA; 8 Section of Infectious Diseases, Department of Medicine, Boston Medical Center & Boston University Chobanian & Avedisian School of Medicine, Boston, MA, USA; 9 Department of Health Law Policy & Management, Boston University School of Public Health, Boston, MA, USA; 10 Evans Center for Implementation and Improvement Sciences, Department of Medicine, Boston University Chobanian & Avedisian School of Medicine, Boston, MA, USA; 11 Center for Integration Science, Brigham and Women’s Hospital, Boston, MA, USA

**Keywords:** heart failure, qualitative, Haiti, low-income country, global health, barriers

## Abstract

*Background:* Heart failure (HF) is a leading cause of hospitalizations in Haiti. However, few patients return for outpatient care. The factors contributing to chronic HF care access are poorly understood.

*Objective:* The purpose of this study is to investigate the facilitators and barriers to accessing care for chronic HF from the patients’ perspectives.

*Methods:* We conducted a qualitative descriptive study of 13 patients with HF participating in three group interviews and one individual interview. We recruited patients after discharge from a nongovernmental organization-supported academic hospital in rural Haiti. We employed thematic analysis using emergent coding and categorized themes using the socioecological model.

*Findings:* Facilitators of chronic care included participants’ knowledge about the importance of treatment for HF and engagement with health systems to manage symptoms. Social support networks helped participants access clinics. Participants reported low cost of care at this subsidized hospital, good medication accessibility, and trust in the healthcare system. Participants expressedstrong spiritual beliefs, with the view that the healthcare system is an extension of God’s influence. Barriers to chronic care included misconceptions about the importance of adherence to medications when symptoms improve and remembering follow-up appointments. Unexpectedly, participants believed they should take their HF medications with food and that food insecurity resulted in missed doses. Lack of social support networks limited clinic access. The nonhealthcare costs associated with clinic visits were prohibitive for many participants. Participants expressed low satisfaction regarding the clinic experience. A barrier to healthcare was the belief that heart disease caused by mystical and supernatural spirits is incurable.

*Conclusions:* We identified several facilitators and barriers to chronic HF care with meaningful implications for HF management in rural Haiti. Future interventions to improve chronic HF care should emphasize addressing misconceptions about HF management and fostering patient support systems for visit and medication adherence. Leveraging local spiritual beliefs may also promote care engagement.

## Introduction

Cardiovascular disease (CVD) is the leading cause of death and disability worldwide with 75% of cardiovascular-related mortality occurring in low- and middle-income countries (LMIC) [1]. In Haiti, CVD has a high disease burden, responsible for an estimated 30% of death [2]. Heart failure (HF) is a major cause of CVD morbidity in Haiti [3, 4] and other LMICs [5–8]. CVD is prevalent in about 15% of Haitian adults [9] and HF is about tenfold more prevalent in Haiti than in the USA [10].

Patients living with HF require chronic care to ensure consistent access to medications, education, and clinicians [11]. Failure of chronic care systems can result in worsening of symptoms, preventable hospitalizations, decreased quality of life, and premature death. At a rural referral hospital in Haiti, only about one-third of patients who had been hospitalized for HF were linked to outpatient care within 30 days [3]. Further, of those patients successfully linked into care, only about half returned for a second outpatient follow up within 60 days of discharge.

Barriers to chronic care for patients with HF have been examined, primarily in high-income countries. Common barriers include self-care challenges such as poor symptom management, medication adherence, and discontinuous access to healthcare facilities [12–14]. Qualitative studies in LMICs of barriers to HF care identified similar challenges, but also revealed unique contextually relevant barriers. For example, specific barriers in Kenya were financial challenges, inadequate social support, and limited disease education [15]. Management of other chronic diseases such as hypertension [16, 17] and human immunodeficiency virus (HIV) suffer from the similar challenges [18]. Such commonalities are useful when considering barriers specific to HF management versus barriers to chronic disease management. Further, there may be unique contextual factors in Haiti affecting HF care.

The purpose of this study is to investigate the facilitators and barriers to chronic care among patients with HF in rural Haiti. Investigating barriers to care requires understanding patients’ ability to self-care and access clinic-based services [14]. Qualitative interviews are a useful means of investigating these experiences by permitting direct questioning of patient attitudes, beliefs, and behaviors, as well as questions about socioeconomic circumstances influencing care [19]. In Haiti, qualitative research has played a key role in the development of health care models that address access [20]. Thus, this study explores the experience of HF in patients in a LMIC using in-depth qualitative methods to expose gaps that must be addressed to improve chronic HF care.

## Methods

### Study setting

Hôpital Universitaire de Mirebalais (HUM) is located in the rural Central Plateau of Haiti. It is a 300-bed academic referral hospital operated in partnership between the Haitian Ministry of Public and Population Health and the nongovernmental organizations Zanmi Lasante and Partners in Health. A low one-time registration fee of about US$1 provides access to inpatient and outpatient care, diagnostic tests, and medications. After hospital discharge, patients with HF are generally provided a 30-day supply of medications and asked to return for clinic follow-up. Patients hospitalized at HUM with HF are mostly women (60%) and present with advanced symptoms, with a 10% in-hospital mortality [3]. While their mean age is 53 years, about one-third are younger than 40 years old.

### Study participants

We recruited patients with HF from HUM for three group interviews. We included adults > 18 years of age who were discharged from HUM with a primary diagnosis of HF. We first screened a total of 53 patients in the hospital. The study research coordinator, who had prior training in qualitative interviewing techniques but was not part of the clinical care team (BDI), introduced the study to potential participants and recorded basic demographic and contact information for them and their family or friends. Linkage from hospital discharge to outpatient clinic-based care within 30 days is a major step along the chronic care cascade. Patients who are either linked or not linked to care within 30 days may face substantively different facilitators and barriers to care. Thus, we a priori wanted to study patients who were and were not linked to care within 30 days. Thirty days after discharge, we identified patients who were either linked or not linked to the HUM clinic by reviewing the medical record. BDI contacted patients by mobile phone to invite them to participate in an interview. Fifteen of the patients (28%) had died during the 30-day period after hospital discharge. We called patients at least three times. Then, if we could not reach them, we called their family or friends. We then purposely recruited participants for interviews to achieve diversity of age, gender, and distance from the hospital. One of the study participants arrived late for the group interview, and we then conducted an individual interview on the same day.

### Data collection

We developed an interview guide to examine patients’ perspectives of the facilitators and barriers to chronic care (supplement). Our goal was to use the study findings to inform future interventions to improve chronic care for patients with HF. The interview guide employed key concepts from literature on access to chronic care services in low-income countries informed by the socioecological model [21]. We pilot-tested the interview guide with three patients with HF at HUM and subsequently revised the interview guide to improve clarity. The final interview guide is provided in the supplement.

We invited between three and six patients to participate in group interviews held in a nonclinical setting at HUM from October 2018 to January 2019. One participant arrived late for the group interview and participated in an individual interview on the same day. In total, we interviewed 13 participants across four interviews. BDI conducted all interviews, first introducing that the study findings would influence future clinical programs. Interview sessions were conducted in Haitian Creole and digitally recorded. The group interviews lasted a mean of 59 min (range 52–70 min). The individual interview lasted 29 min. Afterwards, participants were given light refreshments and a transportation subsidy.

We also collected quantitative data to characterize participants with respect to demographics, socioeconomic status, and clinical severity. We administered a socioeconomic survey adapting the Multidimensional Poverty Index developed by the Oxford Poverty and Human Development Initiative and the United Nations Development Programme [22]. The survey incorporates ten indicators among three dimensions: health (e.g. child mortality and nutrition), education (e.g. years of schooling and attendance), and living standards (e.g. cooking fuel, sanitation, water, electricity, floor material, and assets). Survey questions in Haitian Creole were adapted from the most recent national Mortality, Morbidity and Use of Services Survey 2016–17 (EMMUS) [23]. We extracted clinical characteristic data relevant to HF severity including symptoms (New York Heart Association classification), HF category, echocardiographic findings (left ventricle ejection fraction), and laboratory tests (sodium, creatinine, and hemoglobin) from the hospital record. We recorded descriptive data in a Research Electronic Data Capture database (REDCap, Vanderbilt University, Nashville, TN) [24] hosted at Boston University, CTSI (1UL1TR001430). One patient was not able to complete the descriptive data survey, and some laboratory values could not be found for all patients.

An independent Haitian company transcribed the recorded interviews in Haitian Creole, then translated them to English. BDI reviewed the transcripts in both Haitian Creole and English and edited them for accuracy. Another bilingual team member (M.I.) reviewed discrepant translations.

### Data analysis

As our objective was to investigate the barriers to heart failure care to inform future interventions, we conducted a qualitative descriptive study [25]. Two research team members (E.B. and G.F.K.), who were not involved in the care of the participants, coded the transcript data. We coded the group and individual interviews in the same manner using a combination of open and emergent coding. After each transcript, we discussed and resolved coding discrepancies. We performed content analysis to develop a rich description of the experience of seeking follow-up care for people living with HF [26]. We condensed the codes into themes and patterns relevant to facilitators and barriers to chronic care organized by the socioecological model [21], keeping a detailed audit trail. We achieved theme saturation after analyzing data from the first 10 of the 13 subjects. We used NVivo software to manage the data and assist in code aggregation.

We summarized the quantitative survey data using frequencies, proportions, means, or medians. The multidimensional poverty indicators have defined thresholds to be considered deprived (yes/no). We calculated poverty as the percent of collected variables that were deprived for each person—the intensity of multidimensional poverty [27].

### Ethics

The Institutional Review Boards of *Zanmi Lasante* in Haiti (SN001) and Boston University Medical Campus (H-36222) approved the study. We obtained written informed consent from all participants at the time of the interviews.

## Results

We interviewed 13 patients, including seven women and six men. Participant demographic and clinical characteristics are presented in Table 1. Median age for all participants was 55 years and differed for women (52 years) and men (77 years). Patients were evenly distributed by distance from the hospital across address zones: primary catchment (closest), regional referral, and distant referral (farthest). Poverty intensity was high among participants as they were deprived in a median of four of ten socioeconomic indicators.

**Table 1:** Participant demographic and clinical characteristics.

CHARACTERISTIC	WOMEN	MEN	TOTAL
*N*	7	6	13
Age (years), median (IQR), (*n* = 12)	52 (33.5,53)	77 (74, 79)	55 (49.3, 74.8)
Address zones:			
Primary catchment (closest)	2	2	4
Regional referral	1	3	4
Distant referral (farthest)	4	0	4
Unknown	0	1	1
Poverty intensity, mean (s.d.), (*n* = 9)	40% (6%)	44% (9%)	42% (7%)
NYHA class III or IV on admission, %	100% (7/7)	100% (5/5)	100% (12/12)
LV ejection fraction, %			
Preserved (≥ 50%)	0	0	0
Mid-range (40–50%)	17% (1/6)	0	9% (1/11)
Severely reduced (< 40%)	83% (5/6)	100% (5/5)	91% (10/11)
Creatinine (mg/dL), mean (*n* = 11)	1.8 (1.3)	1.5 (0.5)	1.7 (1.0)
Sodium (mmol/dL), mean (*n* = 11)	145 (7)	155 (2)	149 (7)
Hemoglobin (mg/dL), mean (*n* = 12)	9.9 (2.3)	12.0 (2.0)	10.8 (2.3)
Length of stay, days, mean (*n* = 12)	18.0 (12.4)	17.6 (12.4)	17.8 (11.9)
Linked, %	57% (4/7)	80% (5/6)	69% (9/13)
When data are missing, proportions or number are shown. Poverty intensity: the mean [standard deviation (s.d.)] percent of multidimensional poverty indicators deprived are shown. NYHA, New York Heart Association Linked: patients with clinic encounter within 30 days of discharge			

In this qualitative descriptive study, we categorized themes into domains based on the socioecological model: individual factors, interpersonal/social network factors, organizational/health system factors, and community/spiritual belief factors. We further classified the themes as facilitators or barriers. Participants described their personal successes and challenges with navigating the health system. They also reflected on their observations of friends and family with similar illnesses as these influenced perceptions of their own health and relationship with the healthcare system.

We first summarize the principal facilitators and barriers, followed by reporting detailed findings. Principal individual-level facilitators reflected patients’ experiences with the improvement of their HF symptoms with acute care and desire to maintain this improvement. These positive experiences resulted in trust in the health system. Further, participants described a spiritual world-view inclusive of the health system as an extension of God’s influence. Many patients also reported receiving assistance from their social networks for daily activities and to access care.

**Figure 1:**
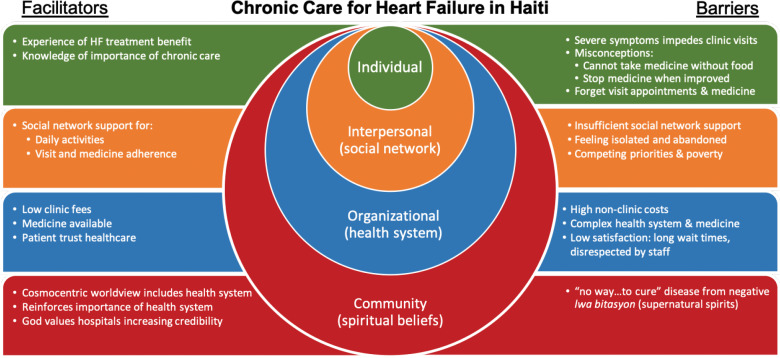
Facilitators and barriers to heart failure chronic care in Haiti organized by the socioecological model.

Principal barriers reported by patients were misconceptions, including needing food to take HF medications and not needing HF medicines when symptoms improve. Patients described insufficient support from social networks, isolation, and abandonment. Health system barriers included high nonclinic costs, confusing health systems, and low satisfaction with the clinic experience. In reflecting on their observations of friends or family with heart disease who did not improve, patients described mystical and supernatural spirits that could not be cured. The major facilitators and barriers are summarized in Figure 1 and detailed in the following sections.

### Individual domain

#### Facilitators: perception of the benefits of acute HF treatment and maintenance care

The improvement in their conditions with HF treatment was a frequent theme across interviewees—all of whom had prior in-hospital care for HF. One study participant noted, “Since I have been taking the medicines, they make me feel much better” (male patient). Further, participants understood the role of health facilities to obtain treatment for severe symptoms. As another participant remarked, “Once you suffer from heart disease you have to go to the hospital because no one else can save you” (male patient).

Continuous engagement with the health system was identified as essential to prevent worsening symptoms. Patients were aware of the importance of maintenance care and its impact on symptoms. As one participant described, “Little by little if you are sick… each time you go to go see a doctor...slowly, you can get better” (male patient).

Patients also prioritized heart disease over other illnesses. When asked about the relative severity of HF, one patient explained, “For me, I believe that heart disease is the hardest, because when it attacks, it hurts” (female patient).

#### Barriers: severe symptoms, misconceptions, and self-care challenges

Occasionally, a patient’s chronic HF symptoms will become uncontrolled. Severe symptoms including shortness of breath and fatigue can make it difficult for a patient to come to the clinic. When asked why they had missed previous clinic appointments, one participant explained, “The reason why I did miss my appointment twice was because my health situation got worse before the appointment date” (male patient).

We identified several misconceptions among study participants. Often, they thought they needed to ingest their HF medications together with food. However, they frequently lacked food and, thus, did not take medications. According to one participant, “Sometimes I spend the whole day without food, and I cannot take the medicines” (female patient). Some participants also reported stopping their chronic medications when their HF symptoms improved. One participant described: “I sometimes don’t take the medicines. Let’s say I have physical pain and I take the medication and the next day it does not hurt anymore, then tomorrow I’m a little negligent” (male patient).

Self-care includes remembering the date of clinic visits or when to take daily medications. Patients reported difficulty remembering to do these activities. When asked, participants explained, “Well, once I missed an appointment because I was late” (female patient). Another noted, “Often it’s because of my work. I sometimes forget” (male patient).

Patients also described difficulty arriving on time for clinic appointments despite their best intentions often attributed to transportation challenges. As one participant explained, “Sometimes you cannot find the tap-tap [public bus] or the motorcycle to come [to clinic]. That is why sometimes it takes longer to get here.” (female patient). Furthermore, patients who arrived late on their appointment day were not seen. “You cannot see the doctor. You have to come the next day because you arrived a little bit late” (female patient). Another patient described “if you are not [at the clinic] they call someone else then…they give you another appointment. In the meantime, your heart is still hurting” (female patient).

### Interpersonal domain: social network

#### Facilitators: support from family and friends

Patients frequently described the essential support they received from family and friends. This support ranged from help with daily activities—such as cooking food—to helping patients come to the clinic. One patient said, “I am sick, I have a heart disease problem, but my children are taking care of me” (male patient). Another patient described the support she has to be able to attend appointments: “Sometimes when I’m coming and don’t want to miss the appointment with the doctor, I ask my friend to help me” (female patient).

#### Barriers: insufficient network support, and competing priorities worsened by poverty

Lack of family and friend support was a barrier to care. Some patients reported feeling isolated and abandoned after becoming sick. According to one patient who felt abandoned, “I have been sick for the past 2 years...no one comes to visit or asks about me” (female patient). Another patient explained that “When it comes to family, they only know you when you own things. When you are poor, they don’t know you...they don’t give me a phone call when they have information about my health” (female patient). Further, coming to the clinic was more difficult for those without a sufficient social network. As one patient explained, “Sometimes it’s not easy for me to come here, because the person that usually helps me does not live in my area” (male patient). Patients also reported many competing priorities for their time and resources which were exacerbated in the setting of poverty. One patient explained, “My children started school, but I had to use their school money to come to the hospital” (female patient).

### Organizational domain: health system

#### Facilitators: low clinic fees, available medications, and trust in the system

Participants stated that fees for clinic visits were extremely low and are not a barrier to clinic-based care. As one participant explained, “When you go to other hospitals, they give you high bills...But here, once you come, they will take care of you” (female patient). Medications in the NGO-supported clinic are more available than other facilities; one participant noted, “every time I come here, I always find all of my medicines” (female patient). Participants also described the trust they have in the health system, according to one participant, “I find help at the hospital, that is the reason why I’m alive today” (female patient). Another patient stated: “It’s here at this hospital where they treat me for the other diseases I have, and I have had improvement for my conditions” (female patient).

#### Barriers: high nonclinic costs, complex health systems and medications, disrespect by clinic staff, and long wait times

While the cost of the clinic visit itself is quite low, there are other costs associated with coming to the clinic. The cost of transportation was frequently described. As one patient stated, “Sometimes the transportation fee prevents me from coming” (female patient). Further, due to the structure of the clinic, patients often had to include travel days before or after the clinic visit with some sleeping on the ground outside of the clinic. Additional travel days resulted in extra costs for lodging and food. In describing a multiday trip, one patient explained, “If you did not bring enough money, you end up not eating that day” (female patient).

Patients described navigating the health system as complex. Patients had to arrive at certain times for their visit. Seeing the providers for urgent issues between scheduled visits was difficult. According to one patient, “I don’t like how it is now, especially when you come, and they ask you to go and come back another day” (male patient). Patients reported their experience in the clinic was poor. They felt disrespected by clinic staff including at registration and by the nurses. One patient said, “The people in the hospital look down on us” (female patient). Patients must endure long waits prior to being seen by doctors and the order was not clear to participants. As one participant explained, “it takes them a long time to call you, I guess because there is a lack of order” (male patient).

Medication regimens were described as complex as patients often take multiple medications several times a day. According to one participant, “I have to take some medications once a day, some twice a day, and some three times a day” (female patient).

### Community domain: spiritual beliefs

#### Facilitators: support from God, God supports the health system

Many patients described how their spirituality influences how they view their own illness and the role of the healthcare system. God was seen as the primary reason for illness and health. Improvement in health is often attributed to God’s will. According to one participant, “If it wasn’t the will of God, I wouldn’t be alive right now” (female patient). Further, when seeking care, participants often seek divine help: “When you are sick you need to ask God for help” (male patient).

Frequently, participants described the hospital or physicians as mediators of God’s will. One respondent noted, “If God did not send this hospital, I can tell you that many people would be dead by now” (female patient). Another patient said: “I came here to the hospital and I found good treatment, but it’s because of God” (male patient).

#### Barriers: negative supernatural spirits cause HF and cannot be cured

While participants overwhelmingly reported their spiritual beliefs to positively reinforce the role of the healthcare system, they did reference the effects of “bad spirits” as a cause of HF in some people. In Haiti, situations attributed to multigenerational spirits known as *lwa bitasyon* may explain current illness [28]. *Lwa bitasyon* can have positive or negative effects on an individual’s life. When asked about their observations of other people with heart disease, one participant explained, HF “sometimes originates from mystical and [super]natural spirits from your *bitasyon*. In this case, you cannot find any treatment to cure it. If it is a *lwa bitasyon* that attacks you and gives you that disease, there is no way you will be able to cure this” (male patient). However, study participants did not report these “spirits” as specific barriers to their own personal care experience.

#### Facilitators and barriers among linked and nonlinked patients

In an exploratory analysis, we evaluated if reported facilitators and barriers to HF care differed on the basis of prior patterns of follow-up. We identified similar themes between patients who had a clinic visit within 30 days of discharge (*n* = 9) and those who experienced a care gap greater than 30 days (*n* = 4). While the numbers of participants in each of these subgroups is small, the consistency of identified themes suggested there may not be substantially different barriers.

## Discussion

This qualitative descriptive study of Haitian adults previously hospitalized with acute HF revealed a diversity of facilitators and barriers to outpatient care. Our analysis identified several potential barriers that will require strategies or facilitators to be leveraged to improve chronic management for patients with HF in Haiti and possibly other LMICs. Ours is the first qualitative study exploring the experiences of patients living with HF in Haiti. While some of the observed facilitators and barriers are expected on the basis of the experiences of patients living with other chronic diseases in LMIC, particularly hypertension [14, 15] and human immunodeficiency virus (HIV) [29], others were unexpected.

We identified several unique and unexpected barriers that may influence future interventions. Several misconceptions expressed by patients may be amenable to educational interventions. For example, many patients mistakenly believed that they could not take their HF medications without food. Food insecurity is highly prevalent in Haiti with nearly half of the population unable to meet basic caloric requirements [30]. Although patients believed that they had to take their HF medications with food, absorption and side effects are generally not influenced by food. However, medications for HIV, in particular, are often better tolerated when taken with food. It is possible that patients believe they should take all medicines with food or that healthcare providers are unintentionally miseducating patients. A study of patients with HF in Uganda revealed a desire for more information about self-care [31]. Strategies incorporating education of both patients and providers may address this misunderstanding.

Though there are few studies of chronic HF care experiences in LMIC, there is a robust literature exploring barriers to care for other chronic diseases including hypertension [14, 15] and HIV, including in Haiti. It is appropriate to compare patient experiences of HIV with HF as both require chronic management and have a high morbidity and mortality if untreated. Further, it is useful to consider the implications of our research in Haiti with chronic disease interventions that have been attempted in other LMICs, given the common barriers that exist in low-resourced settings.

The patient experience of caring for an acute condition has some similarities with chronic conditions. Medications may lessen symptoms and alleviate suffering, and follow-up appointments may be needed to ensure improvement. However, unlike for acute conditions, medications for chronic HF must be taken daily to prevent symptom worsening or recurrence even if patients are feeling well. Participants reported difficulty with remembering to take their medications, as is also seen among people with hypertension [16, 17]. Our findings were similar to other studies of patients living with another chronic condition—HIV. Participants perceived a greater need to take medications if they also experienced symptoms such as shortness of breath [32]. Among patients living with HIV, improved health was motivating for engagement in chronic care [29, 33]. However, persistent symptoms, despite medications, should not be viewed as a failure of therapy [34]. Interventions to improve long-term HF outcomes will need to focus on education or other adherence support systems, as have been widely implemented for HIV care [29].

Reliance on social networks for support was an important theme observed in our study and studies of other chronic conditions [32–35]. Support from family and friends was instrumental to helping participants maintain appointments and adhere to therapy. They may have accompanied patients to healthcare facilities for appointments or encouraged compliance with medications. Participants in our study without support from family or friends described substantial difficulty in attending regular clinic visits. It is possible that improved control of symptoms or physical conditioning may alleviate some of the need for assistance in attending scheduled appointments.

Experiences of health system interactions were similar to studies of other conditions in diverse settings. Participants in our study endorsed an appreciation of the role health system for acute care to improve symptoms and chronic care to maintain health. Among people living with HIV, similar health improvements have led to high levels of trust in health systems and clinicians [32, 36]. Though our participants reported feeling disrespected by some staff members, they reported having trust in the doctors treating them. Developing trust in clinicians may overcome a lack of knowledge about the long-term nature of chronic conditions [37].

Despite prior positive experiences with respect to improved health and trust, participants described an overall poor experience at the clinic. Long distance and high transportation costs were also barriers among patients living with HIV [33, 38]. Among participants coming to this clinic, many often slept on the ground outside of the clinic to be seen the following morning. Patient satisfaction can influence long-term care adherence. Despite trust in the hospital, a lack of respect from the staff was a barrier frequently described by study participants. Feeling disrespected is reported in other departments of the same hospital and in many other LMIC health facilities. For example, women coming to this same hospital for labor and delivery reported perceiving lack of respect as hospital staff criticized their appearance or made jokes [39]. Similarly, hypertension patients in Kenya often avoided coming to clinic due to fear of “harsh” treatment by clinicians [16], whereas unprofessional and discourteous staff deterred adherence to care among people living with HIV [32]. In Zambia, patients living with HIV were willing to wait an extra 9 h or travel 45 km further to get care from nice, rather than rude, clinicians [40]. Despite some feeling disrespected during their clinic encounter by ancillary and nursing staff, participants still reported a high degree of confidence in their physicians’ ability to treat their health conditions.

Emotional factors, such as stigma, can impact chronic disease care. Stigma is well-characterized as a barrier to care for people living with HIV [41]. Further, a study in Uganda identified stigma as a major barrier to care among women of child-bearing age with rheumatic heart disease as they were told they could not give birth resulting in social isolation [42]. When seen taking their medication, these Ugandan women reported being accused of having HIV. While study participants reported feeling isolated and abandoned, the role of stigma was not specifically described. This could be because our participants were generally older with only two female participants of child-bearing age.

Nearly all of the participants related their interactions with the health system to their spirituality. In Haiti, a “cosmocentric” perspective is dominant and part of the Vodou legacy compared with an “anthropocentric” view of health and disease [43]. In the cosmocentric view, individuals exist as part of a larger universe of familial and divine spirits, ancestors, social relationships, and the natural world. Conversely, individuals view themselves at the center, controlling their own universe in the anthropocentric perspective. Participant’s cosmocentric perception and its influence on interactions with the health system was a persistent theme throughout the interviews. Subjects frequently described that spiritual forces were the source of either their illness or their good health. Additionally, participants described that clinicians and the hospital were mediators of God’s influence in their lives. Our observations were consistent with prior studies in Haiti.

In Haiti, people may consider “natural” versus “supernatural” sources of illness. Supernatural illness may be related to *lwa bitasyon* and breaches of the connections between the person, God, nature, and community [43]. Beliefs in Vodou and other “supernatural” sources of illness may lead patients first toward traditional practitioners who are better able to “manipulate the spirit” and away from doctors and western health systems [44]. This belief system may restrict the use of hospitals and medications [45, 46]. A study from sub-Saharan Africa found that health beliefs may have influenced the preferred source of healthcare or led to delays in reaching appropriate treatment [34]. Among study participants—who were all patients who had engaged in acute hospital-based care—no one reported seeking alternative healthcare sources or traditional healers. It is possible that these belief structures may have influenced initial care but not for chronic care among those who had been previously hospitalized. Further, patients verified that their cosmocentric views improved their degree of trust in doctors and health facilities and facilitated chronic care engagement.

The facilitators and barriers we identified through this analysis and organized by the socioecological model can be used to inform future interventions to improve chronic HF care in rural Haiti and possibly other LMIC. Education interventions targeting patients may be able to address the identified misconceptions. Systems to remind patients of clinic visits and better connect patients to the clinic may be beneficial. Interventions can leverage the facilitators to further engage social networks and emphasize prior positive care experiences. Trust in the health system can be reinforced by aligning the work of healthcare staff and clinical facilities as components of a broader universe combining the divine and natural worlds. Developing future interventions will require engagement of a broad group of stakeholders: community members, community health workers, and clinicians. Improving health system responsiveness and the patient experience will require changes in healthcare worker attitudes and behaviors. Additionally, patients hospitalized for HF often have very poor prognoses. Among patients screened for this study, about one-quarter died within 30 days. Thus, targeted interventions to improve linkage to care earlier than 30 days after hospital discharge may be needed for some patients. Though challenging, palliative care for patients with severe HF may need to be incorporated into health systems [47].

Our study has a number of limitations. First, our study has a sample of 13 participants. While we achieved thematic saturation prior to the last group interview, it is possible that other barriers may have been uncovered had we interviewed more participants. Relatively small sample sizes are consistent with other qualitative descriptive studies [48]. Second, our study suffers from selection bias against patients who are more impoverished. We recruited only patients with working telephones. In addition, even though we provided a transportation subsidy, we interviewed only patients who were able to find or afford a ride to the clinic. Third, we included only patients with HF who came for initial care to the hospital. There may be other key barriers that were not captured among patients who did not come for initial care at the hospital. These barriers may be relevant for chronic HF care. Fourth, the linked and not linked subgroups were both small. However, there was no meaningful difference in endorsed themes between the two groups. Fifth, we were unable to invite some patients for an interview because they died within 30 days of hospital discharge. These patients who died may have faced unique barriers to chronic care. Sixth, the patients we interviewed were older, on average, than patients who are hospitalized. It is possible that younger patients may not have been willing to participate in interviews as they may have been working. Seventh, while we intended for all subjects to participate in group interviews, we interviewed one patient individually because they arrived after the group interview was completed. This individual interview may have yielded biased data as the participant would not have the opportunity to react to the experiences of other participants. However, the themes endorsed during the individual interview were aligned with the other group interviews. Eighth, our results may not be generalizable to other LMIC settings though some findings are likely transferrable.

## Conclusions

Individuals with HF in rural Haiti experience a diverse array of facilitators and barriers to accessing chronic outpatient care. While many identified facilitators and barriers are similar to the care of people with other chronic conditions, some are unique to HF and the Haitian context. Understanding patients’ experiences engaging in chronic care can inform patient-centered and culturally inclusive interventions. Future interventions to support HF chronic care in Haiti should emphasize facilitators to care, such as existing social supports and patient trust in health systems. Specifically, improving patient’s health literacy to understand that medications help prevent recurrence of symptoms and can be taken without food, improving the experience of a clinic visit, and decentralizing healthcare systems can address some of the identified barriers. Future interventions must also address barriers to care, including misconceptions about HF management and poor patient satisfaction in clinic interactions. Further research is needed to facilitate our understanding of the scope of challenges to chronic HF care in rural Haiti from multiple perspectives.

## Data Availability

The data that support the findings of this study are available from the corresponding author, G.F.K., upon reasonable request.

## Additional Files

The additional files for this article can be found as follows:

Supplementary File 1. Interview Guide.

Supplementary File 2. Standards for Reporting Qualitative Research (SRQR) Checklist.
