# Supplementary material for: Heart Failure Care Facilitators and Barriers in Rural Haiti: A Qualitative Study
Source: Ann Glob Health. 2024 Sep 25;90(1):60. doi: 10.5334/aogh.4521 (PMC11428663; doi:10.5334/aogh.4521)
Supplement: Supplementary File 1. — Interview Guide. [file agh-90-1-4521-s1.pdf]

Interview Guide for "Heart Failure Care Facilitators and Barriers in Rural Haiti: a Qualitative Study"

Introduction and welcome

We have asked for you to come here today to help us better understand what it is like to be a patient with a disease called heart failure. You are being asked these questions because you have all been treated in the hospital for heart failure at some time. Your answers will help us make improvements in the system to better serve people like you.

I am (facilitator name here), and I will be helping to facilitate this group interview. You have agreed to participate in a study. In this study, we will ask you a series of questions and record your answers. The interview should take about 1 hour. Please answer the questions as best you can. There are no "right" or "wrong" answers. Please give detailed answers so we can best understand your perspective. To try to maintain confidentiality, we as that you not use your name during the interview. All of the information we discuss today will be confidential

Individual Domain: Knowledge, Attitudes, Values, Beliefs, and Goals

In this first section, we would like to learn about your understanding of the disease called heart failure. We will ask you a series of questions related to how HF affects people and the care you receive.

1. I would like to start by asking, what do you know about HF? Is HF a serious problem? Why or why not?
2. How does a person know if he or she has HF? Can someone feel it and what does it feel like to have HF?
3. How does HF affect your life? Is your life different now that you have heart failure?
    a. For example, does it affect your ability to work, to go to school or to do other activities?
4. We are curious about what you know about the treatment of HF. Is heart failure a problem that can be treated and go away for good? If yes, how? If no, can you explain. How do you or others you know it needs to be treated?

Interpersonal domain: social networks family/peers, emotions/habits, healthcare workers and Media

In this second section, I will ask you questions about people who help you, the challenges of coming to the clinic, and your habits related to medication.

5. Think about someone you know who has HF. You may not know anyone else. But if you do, in what way does he/she struggle with their treatment?
6. This next question is specifically about your family and friends. Sometimes people have family or friends that help them when they are sick. How have members of your family or friends helped you with health issues? What kind of help do you need or get? Do you always get as much help as you need? Have family or friends helped you go to the hospital or clinic?
7. Patients with heart failure may need routine visits with a doctor or nurse. The next question has to do with your visits to your doctors or nurses.

    a. Sometimes it is hard to get to hospital appointments. Are there things that make it difficult to make it to your appointments see a nurse or doctor? Can you give examples of a time that you missed an appointment?
8. The next questions are about how you get to the hospital for your medical appointments. We are interested in understanding what is it like for you to get back and forth to the clinic and if there are things that could make it easier.
    a. How long does it take you go come to the clinic and return home?
    b. How much does it cost to go to and from the clinic?
    c. Do you struggle paying for other things if you go see your nurse or doctor?
    d. Do you have to give up other things (such as food, shelter or other activities) to see your nurse or doctor?
    e. If you did not have to pay for transportation, do you think you would come to the clinic more frequently?
9. Often, people like you with heart failure have to take many medications each day, which can be difficult. Some people may feel that their medications are helpful, while others may say that the medications make them feel unwell. How do you manage with your medications? How do your medications affect you? Do you take medications once per day or multiple times per day? Do you forget to take your medications on occasion? Why?

Organizational/Health system domain: (Accessibility, Availability & Affordability): Programs & Services Skills, Memory, Money & Time, and Facilities

In this last section, I will ask you questions about the health system, the way it supported you through getting access to good quality healthcare, as well as the way it can be improved.

10. Some clinics and hospitals charge a different amount of money for healthcare.
    a. How important to you is cost when deciding which clinic or hospital to go to?
    b. Do you think that the cost of care was high at the hospital you went to for care?
    c. Do you feel you get good care for the money you pay?
11. Think of the last time you needed to pick up medication. Describe how you got your medication? What was that like for you? Did the pharmacy have the medication at that time? Did you have to come back to the same pharmacy at a later time? Did you have to go to a different pharmacy?
12. Some people with other diseases have a trained community member help with assistance at their home. We are thinking that we can offer similar services for people with heart failure as well. What would it be like for you to have a trained worker help you at home? What types of things do you think the community member can help with?
13. For some people with other diseases, we call them on their phone to check them periodically and remind them about appointments. We are thinking that we can offer similar services for people with heart failure as well. What would it be like for you to receive a follow-up phone call after you leave the hospital? What types of things could be discussed over the phone?
14. The next questions are about your experience with the health care system. This is an opportunity for you to tell us what we could do to make the healthcare system work better for you.

a. What advice do you have for clinics, doctors, nurses, and community health workers about how to be more helpful to you in treating your heart failure?
b. What kind of things would make your visit more pleasant? For instance, do you feel you wait too long at the clinic to see the doctor?

15. Wrap Up. Do you have any questions or concerns about your participation in the study today?

Thank you for your participation. Reminder that all information discussed today will remain confidential.
